# Proteomics analysis of water insoluble-urea soluble crystallins from normal and dexamethasone exposed lens

**Published:** 2011-12-28

**Authors:** Lin Wang, DongRui Liu, Ping Liu, YongBin Yu

**Affiliations:** 1 Eye Hospital, The First Affiliated Hospital, Harbin Medical University, Harbin, China; 2 Key Laboratory of Harbin Medical University Eye Center, Harbin, China

## Abstract

**Purpose:**

The aim of this study was to identify glucocorticoid induced cataracts (GIC)-specific modified water insoluble-urea soluble (WI-US) crystallins and related changes after rat lens were exposed to dexamethasone (Dex).

**Methods:**

We separated WI-US lens proteins by two-dimensional electrophoresis (2-DE). The crystallins were then analyzed with matrix assisted laser desorption/ionization time-of-flight tandem mass spectrometry (MALDI-TOF-MS/MS). Protein levels and morphological changes of αA- and αB-crystallins were also determined. Electronic microscope of lens and native-page analysis of crystallins were further determined.

**Results:**

Measured masses, isoelectric points (pIs), and amino acid sequences of all detected crystallins matched previously-reported data. Analysis by 2-DE indicated that αA- and αB-crystallin increased when the lens was viewed under 1 µM and 10 µM Dex, which was identical with the results of western-blot, immuno histochemistry or fluorescence; βB2- and βA3-crystallin increased when lens was viewed under 1 µM Dex and 100 µM Dex. βA1-, βA4-, and βB1-crystallins decreased under 0.1–100 µM Dex. Electronic microscope figures showed the condition of the lens center gradually worsened and cracked between fiber cells that became larger under 1–100 µM Dex. Moreover, αA-crystallins were associated with increased phosphorylation (PI decreased).The newly protein spots: βA2-, βA3-, βB1-, and γs-crystallin appeared under 0.1–100 µM Dex. Native-page showed α-crystallin increased when the lens was exposed to 1 µM Dex; however, β-crystallin did not decrease under 0.1–100 µM Dex. The percentage of α-crystallin gradually decreased, however β-crystallin gradually increased, perhaps because the emergence of newly appeared β-crystallin under Dex.

**Conclusions:**

Our results showed multiple WI-US crystallins may be more vulnerable to glucocorticoid stress because of diminished important roles, which will in turn provide a mechanism for GIC from a proteomics perspective.

## Introduction

Posterior subcapsular cataract (PSC) is mainly a complication for patients who accept long-term systemic treatment with steroids [1,2]. Recently, several papers have reported ophthalmological complications induced by glucocorticoids [3-5]. Although there are various studies on glucocorticoid-induced cataracts (GIC), the precise molecular mechanism involved in the formation of GIC has not yet been elucidated because procuring human samples is difficult. Based on some studies on animal models to examine the effects of glucocorticoids [6,7], it takes more than 8 months to produce phenotypic changes with the in vivo model system. In our report, glucocorticoid-induced cataracts were simulated in an ex vivo model, in which rat lenses were incubated with dexamethasone. Opacity was induced in the posterior region of the rat lenses and was morphologically similar to human steroid-induced cataracts.

Properties of crystallins are very important in maintaining the transparency of the eye lens. Many papers have reported that a normal lens leads to the modification of post-translationally proteins, such as mixed disulfide formation; glycation; cross-linking by UV, transglutaminase, or disulfides; phosphorylation; deamidation; and proteolysis [8-14]. Because crystallins undergo very little turnover after synthesis [15], we hypothesize that modifications of crystallins may contribute to cataract by causing activation or degradation of crystallins. Because it also remains unclear how the relative mechanism of water insoluble-urea soluble  (WI-US)  of lens crystallins of lens crystallins during cataract development differs from the normal and glucocorticoid induced cataract lens, we further studied changes of WI-US crystallins after lenses were exposed to four concentrations of dexamethasone (Dex).

Furthermore, the precise protein modifications that occur in the lens after it is exposed to Dex have not been systematically explored; two-dimensional electrophoresis (2-DE) is capable of simultaneously resolving complex mixtures of modified crystallins. These resolved crystallins can then be quantified by image analysis, and posttranslational modifications on excised spots can be determined by mass spectrometry (MS/MS).Thus, in our study, reproducible rat lens WI-US crystallins 2-DE maps were created for lenses of increasing concentrations of Dex to serve as reference maps. Further, the molecular identity of the separated proteins was confirmed by MS/MS, and posttranslational modifications were identified. In future studies, these data will facilitate the identification of specific modifications in cataract lenses of rats induced by Dex.

We undertook the present study to analyze the species present in WI-US proteins of normal lens and cataract lens induced by concentrations of Dex to distinguish those species that were cataract-specific induced by Dex.

## Methods

### Reagents

Dex was dissolved in dimethyl sulfoxide (DMSO) as a stock solution (200 mM). All reagents were from Sigma, St. Louis, MO.

### Preparation of rat lens and drug treatments

Sprague-Dawley male rats (21-day-old) were provided by Animal Laboratories (Harbin Medical University, Harbin, China). Animals were handled in strict accordance with the Association for Research in Vision Ophthalmology Statement on the use of Animals in Ophthalmic and Vision Research. Rats were killed, and the lenses were dissected and flash frozen until use. All experiments using animals conformed to the ARVO Statement for the Use of Animals in Ophthalmic and Vision Research. Rat lenses were carefully dissected from the surrounding ocular tissue and were immediately transferred into the culture in medium 199 (pH 7.2; Sigma), containing BSA (BSA; GibcoBRL, Grand Island, NY) and an antibiotic solution (GibcoBRL; 100 U/ml penicillin, 100 µg/ml streptomycin, and 0.25 µg/ml amphotericin B). Dex was added to final concentrations of 0 µM, 0.1 µM, 1 µM, 10 µM, and100 µM then added to the medium. Each Dex concentration group contained 6 lenses. Lenses were cultured for 48 h at 37 °C under 5% CO_2_. The treatments were the same for all assays. The lenses were cultured and observed daily for 2 days and photographed under a stereomicroscope to record the development of opacity.

### Isolation of WI-US lens crystallins

Each frozen lens was ground in liquid nitrogen and then dissolved in 1 ml of lysis buffer (20 mM sodium phosphate, pH 7.0, 1 mM EGTA, 100 mM NaCl, 1 mM PMSF, 1 μg/ml aprotinin, and 1 μg/ml leupeptin). Lens homogenates were centrifuged at 20,000× g for 45 min at 4 °C and supernatants were removed and collected. The supernatants were designated as water-soluble (WS) protein fractions, and the pellet was designated as the water-insoluble (WI) protein fraction. The WI protein fraction was suspended in a buffer (50 mM Tris-HCl, pH 7.9, containing 6 M urea and 5 mM dithiothreitol) and homogenized. The supernatants were designated as the WI-US protein fraction. The pellet was designated as the water-insoluble-urea-insoluble (WI-UI) protein fraction. The process was repeated twice to recover the WI-US and WI-UI protein fractions, and then combined with the above similar fractions. The WI-US protein concentration was determined according to the Bradford method.

### 2DE and protein identification by MALDI-TOF mass spectrometry

WI-US protein samples were processed by 2D clean-up kit then re-dissolved in a rehydration solution with immobilized pH gradient (IPG) buffer (8 M Urea, 2% CHAPS, 0.5% IPG buffer, 0.002% Bromophenol blue). Isoelectric focusing was performed using IPG gel strips (13 cm, pH 3–10), which were loaded with 300 ug of protein. Spot-picking and digestion were performed using preparative gels, and the gels were stained with Coomassie brilliant blue. After the preparative gels were scanned, protein spots of interest were excised from the gels and digested with trypsin. The tryptic peptides were extracted and dried completely by centrifugal lyophilization. The spots representing differential proteins were first identified by image analysis, and then further analyzed by matrix-assisted laser desorption/ionization-time of flight-tandem mass spectrometry (MALDI-TOF-MS/MS).

MALDI-TOF mass spectrometry and tadem TOF/TOF mass spectrometry were performed using a 4800 MALDI-TOF-TOF instrument (Applied Biosystems, Foster City, CA). Combined mass and mass/mass spectra were used to interrogate rat sequences in the International Protein Index database using the Mascot database search algorithms. Confident protein identification had a statistically significant protein score and the best ion score.

The Coomassie Blue–stained gel images (containing 3D views) were captured and image analysis was performed on a computer (ImageMaster 2D Platinum 6.0 software; GE Healthcare, Piscataway, NJ) to determine the percent at each spot that contributed to the total protein on the gel.

### Western-blot

WI-US protein samples were collected from lenses after treatment with 4 concentrations of dexamethasone then treated with 5× SDS page buffer. Protein concentrations were determined using a bicinchoninic acid (BCA) assay (Pierce, Rockford, IL). Samples were electrophoresed on 12% Tris-glycinepolyacrylamide gels using a commercial apparatus (BIO-RAD Mini-PROTEAN Tetra cell; Bio-Rad Corporation, Hercules, CA) and then blotted on nitrocellulose membrane (Pall-Gelman, Ann Arbor, MI) using a transblot cell (BIO-RAD). Membranes were blocked in 3% BSA and probed with primary antibodies: a monoclonal αA -crystallin antibody (B-2, 1:1,000; Santa Cruz Biotechnology, Santa Cruz, CA), a polyclonal αB -crystallin antibody (FL-175, 1:1,000; Santa Cruz Biotechnology). After washing, the membranes were then incubated with horseradish peroxidase-conjugated secondary antibody (1:5,000; Zsbio, Beijing, China) by standard protocols and visualized by diaminobenzidene (DAB; Tiangen Biotech Co. LTD, Beijing, China) stain using a gel documentation apparatus (FUJIFILM LAS-3000; Fujifilm Corporation, Tokyo, Japan). As an internal control, the membranes were re-probed with a 1:1,000 dilution of mouse anti-rat actine mAb (Beyotime Institute of Biotechnology, Jiangsu, China) using the same conditions described above. Images were captured as described above, and densitometric analyses were performed using an NIH Image (ver.163) with values normalized to the actine signal for each sample and expressed as multiples of increases (or decreases) relative to control samples (developed by Wayne Rasband, National Institutes of Health, Bethesda, MD).

### Analysis of immuno-histochemistry

At the end of the culture period, the nuclear regions of the lenses were placed in phosphate-buffered saline (PBS) and fixed overnight in 4% paraformaldehyde. The lenses were dehydrated, embedded in paraffin, and cut into 3-µm sections. For histology, de-waxed paraffin sections were washed with Tris-buffered saline (TBS; 10 mM Tris and 150 mM NaCl [pH 7.4]) and incubated for 60 min in TBS containing 0.05% Triton X-100, 2% BSA; the sections were then incubated overnight with primary antibodies: a polyclonal αB-crystallin antibody (FL-175, 1:50; Santa Cruz Biotechnology). Afterwards, samples were washed with Tris-buffered saline then incubated for 1 h with horseradish peroxidase -conjugated secondary antibody (1:100; Zsbio, Bejing, China). Histologic images were photographed with a microscope (Eclipse TS100-F; Nikon, Tokyo, Japan).

### Analysis of immuno-Fluorescence

At the end of the culture period, the nuclear portions of the lenses were frozen and cut into 5-µm sections. After being fixed with acetone, lens sections were washed with Tris-buffered saline (TBS; 10 mM Tris and 150 mM NaCl [pH 7.4]) and incubated for 60 min in TBS containing 0.05% Triton X-100, 2% BSA. The sections were then incubated overnight with primary antibodies: a monoclonal αA-crystallin antibody (B-2, 1:50; Santa Cruz Biotechnology), a polyclonal αB-crystallin antibody (FL-175, 1:50; Santa Cruz Biotechnology). Afterwards, samples were washed with Tris-buffered saline then incubated for 1 h with Cy3-conjugated or FITC-conjugated secondary antibody (1:100; Beyotime Institute of Biotechnology). Histologic images were photographed with confocal-fluorescence microscopy (CFM, Leica TCS SP5; Leica Microsystems Ltd, Wetzlar, Germany) or with a fluorescence microscope (Nikon, Eclipse TS100-F; Nikon Corporation, Tokyo, Japan).

### Morphologic changes of apoptotic cells detected by electronic microscopy

For electron microscopy (EM) studies, the nuclear portions of the lenses were fixed in 4% glutaraldehyde (0.1 M sodium phosphate buffer, pH 7.4) and postfixed in 1% osmium tetroxide. The tissues were washed and dehydrated through a graded series of ethanol, cleared in propylene oxide, embedded in Epon mixture, and sectioned at 1 µm and 90 nm with an ultramicrotome. Semi-thin sections (1 µm) were stained with toluidine blue for light microscopy. Thin sections (90 nm) were stained with uranyl acetate and lead citrate, and the super thinly sliced structures of lenses were clearly observed by transmission electron microscopy (H-7650 JEM-1200EX; Japan Electron Optics Laboratory, Tokyo, Japan), then photographed and recorded.

### Native-PAGE of WI-US crystallins

After isolating the WI-US protein from every lens group, we used Native-PAGE to detect expressional changes and percentage of α-, β-, and γ-crystallins in each group. WI-US protein samples were collected from normal lenses and from post-treatment lenses, with 4 concentrations of dexamethasone then treated with 2× native page buffer. Protein concentrations were determined using the bicinchoninic acid (BCA) assay (Pierce, Rockford, IL). Samples were electrophoresed on 6% native Tris-glycine polyacrylamide gels using a commercial apparatus (BIO-RAD Mini-PROTEAN Tetra cell) and the gels were stained with Coomassie brilliant blue.

### Statistics

Data are shown as mean±SEM from triplicate samples. Each experiment was performed at least 3 times to confirm reproducibility. Statistical analysis was performed using ANOVA with Dunnett’s as post-hoc. A p-value of less than 0.05 was considered statistically significant. Statistical software, SPSS 17.0 (SPSS Inc. Chicago, IL), was used.

## Results

### Induction of PSO by dexamethasone

**Figure 1:**
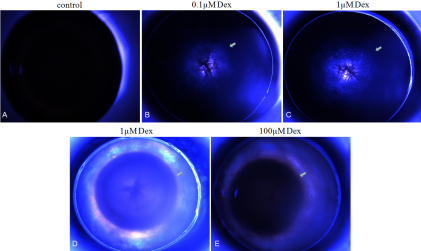
Effects of 4 concentrations of dexamethasone on lens transparency. Whole lenses were cultured for 48 h without (**A**) or with (**B**-**E**) dexamethasone. Photographs were taken at the focus of the posterior zone, or through the anterior pole. In the presence of dexamethasone, posterior subcapsular opacities developed in the lenses. Without dexamethasone, lenses remained clear. Arrows: areas of opacity.

To examine the effects of glucocorticoid on the lens, we developed an ex vivo rat lens model. We isolated rat lenses and treated them in organ culture to cause steroid-induced cataracts directly. The lenses were incubated with 0, 0.1, 1, and 10 µM Dex, and morphologic changes in the whole lenses were recorded photographically with a stereomicroscope. Lenses with posterior opacity were selected. The opacity first appeared at 48 h in the posterior region of lenses incubated with every concentrations of dexamethasone. The opacity (Figure 1B-E) in those lenses incubated with 10 or 100 µM Dex for 48 h were most obvious, whereas they were not detected in the anterior or equatorial regions. Untreated control lenses remained transparent for 48 h treatment of dexamethasone (Figure 1A).

### Differential analysis of protein levels and spot identification

**Figure 2:**
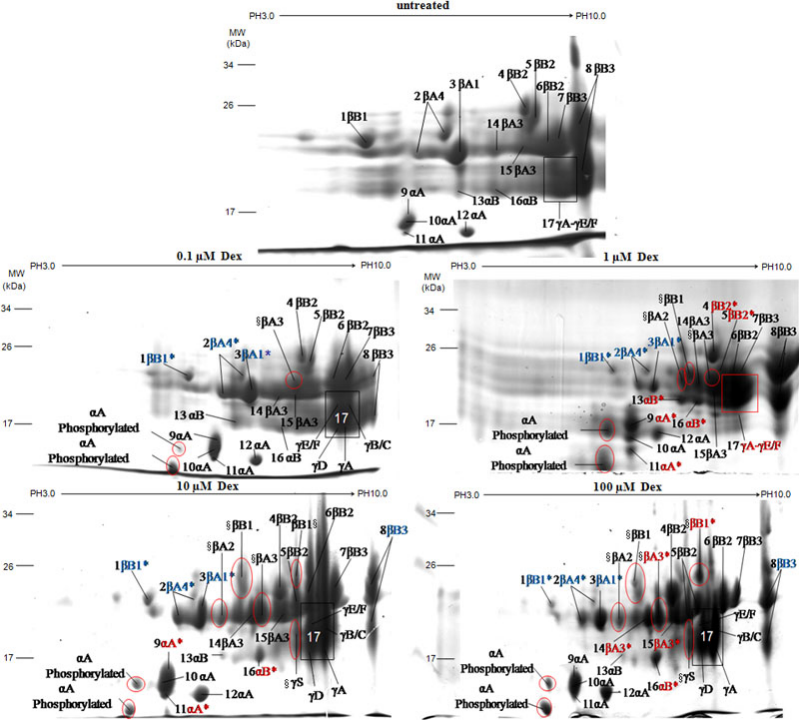
2DE gel image of the total proteome in the normal rat lenses and lenses exposed to 0, 0.1, 1, 10, and 100 µM Dex. The proteins were separated by IEF and SDS–PAGE and stained with Coomassie brilliant blue. These spots were excised and analyzed by MALDI-TOF-MS/MS mass spectrometry. Protein spots that were successfully identified were marked with numbers in this preparative gel image. Increased intensity of protein spots were marked with red words and decreased intensity was marked with blue words, in which significant changes were marked with a red * or with a blue *. Intensity of protein spots that did not increase or decrease were marked with black words. Newly appeared protein spots were marked with a §.

**Figure 3:**
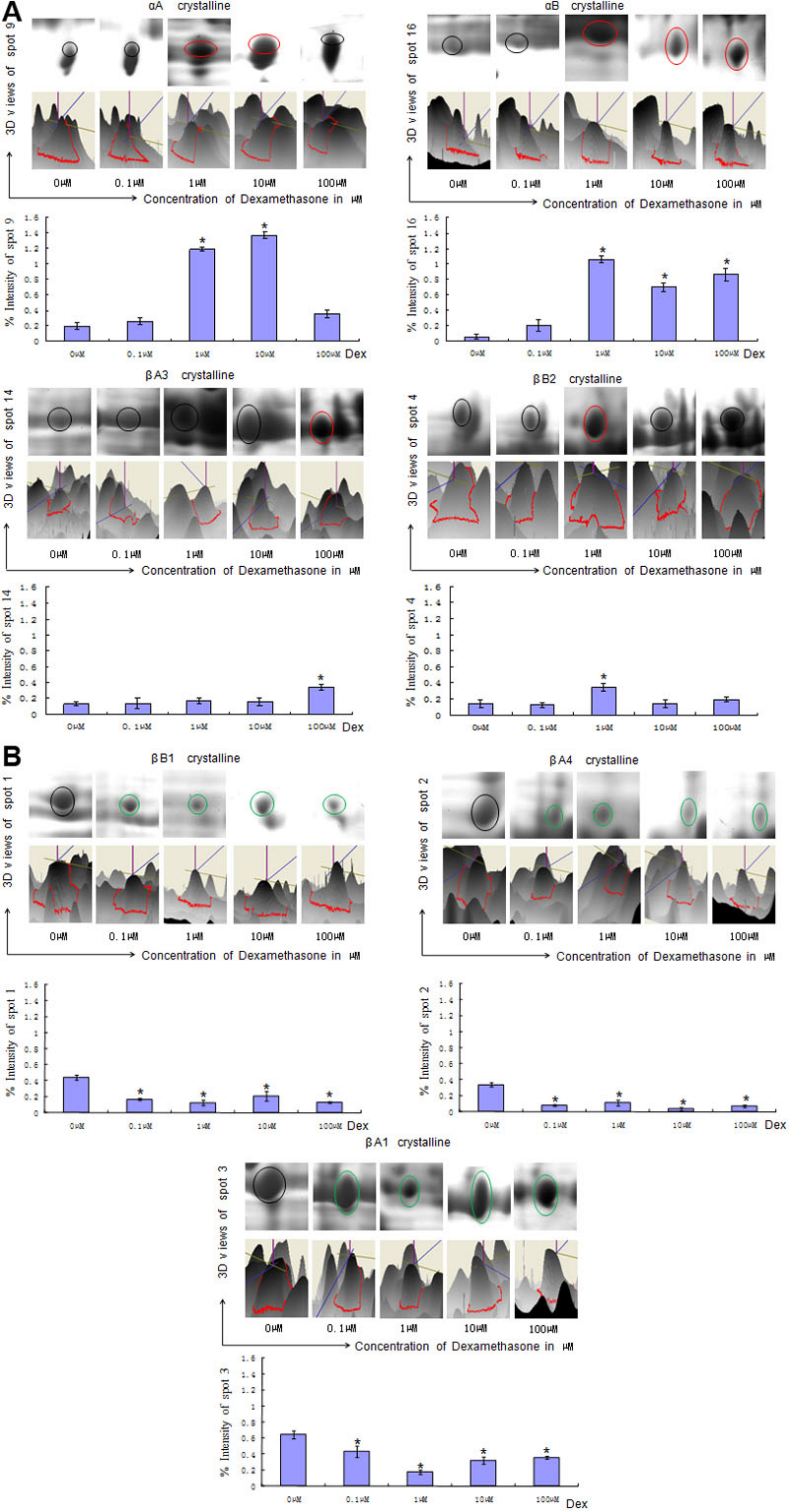
2DE images and three dimensional views of protein spots with significantly different levels; 2DE images, the corresponding three-dimensional views, and histograms of the spots were presented. Intensities of protein spots that increased were marked with a red outline. Decreased were marked with a blue outline, and no significant changes were marked with a black outline. The spots were identified as follows: αA-crystallin (spot 9), αB-crystallin (spot 16), βA3-crystallin (spot 14), βB2-crystallin (spot 4), βA1-crystallin (spot 3), βA4-crystallin (spot 2), βB1-crystallin (spot 1; *p<0.05, n=3).

**Figure 4:**
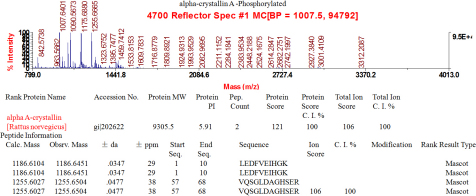
Identification of rat phosphorylated-αA-crystallin, by mass spectrometry of the protein spot digested with trypsin. Amino acid sequences of tryptic peptides recovered from the protein spots were presented in two-dimensional gel electrophoretic maps of water- insoluble-urea-soluble protein fraction, isolated from the lens after exposure to variable concentrations of Dex. The amino acid sequences reported are the tryptic fragments from individual spot, and they did not alter compared with amino sequences of proteins published in PubMed. Thus, we present the MS spectra (PMF spectra) of it.

To investigate the crystallin changes in dexamethasone exposed lenses, 2DE mapping was performed (Figure 2). A greater difference in the amount of protein and a *t*-test score of less than 0.05 was considered to be significant. The peptides representing these spots were analyzed by MALDI-TOF mass spectrometry, and 25 spots were successfully identified (Table 1). A total of 7 protein spots were significantly different between normal and dexamethasone exposed lenses (Figure 3), and each was more or less abundant in the dexamethasone exposed lenses compared with the normal ones. The amino sequences of phosphorylated-αA-crystallin were not altered compared with amino sequences of proteins published in PubMed, and we present the MS spectra (PMF spectra) of it (Figure 4). Based on these analyses, seven unique proteins had different levels between normal and dexamethasone exposed lenses: αA-crystallin (spot 9), αB-crystallin (spot 16), βA3-crystallin (spot14), βB2-crystallin (spot 4), βA1-crystallin (spot 3), βA4-crystallin (spot 2) and βB1-crystallin (spot 1). For each unique protein, the seven spots were examined using 3D (Figure 3). We detected the differences of the intensities of the protein spots. As shown in Figure 3, which suggested that three unique proteins (βA1-crystallin, βA4 –crystallin and βB1-crystallin) were less abundant in the dexamethasone exposed lenses than in the controls (p<0.05). The intensity of proteins αA-crystallin, αB-crystallin tended to increase when lenses were exposed to 1 µM and 10 µM Dex (p<0.05); βB2-crystallin tended to increase when lenses were exposed to 1µM Dex (p<0.05); βA3-crystallin tended to increase when lenses were exposed to 100 µM Dex (p<0.05). The intensity histograms of modified protein spots were related to their overall intensity.

**Table 1:** Proteins identified by MALDI-TOF and tandem TOF/TOF mass spectrometry.

**Spot number**	**Gene name/protein**	**Accession number**	Protein score*	**Prep count**	**Mr**	**Pr**
1	CRYBB1/βB1-crystalline	gi|203623	329	9	28.013.5	8.59
2	CRYBA4/βA4-crystallin	gi|13928956	109	11	22595.7	5.9
3	CRYBA1/βA1-crystallin	gi|149053478	216	4	21392.6	6.1
4	CRYBB2/βB2-crystallin	gi|3127918	332	5	23,294.3	6.5
5	CRYBB2/βB2-crystallin	gi|3127918	405	5	23,294.3	6.5
6	CRYBB2/βB2-crystallin	gi|3127918	398	5	23,294.3	6.5
7	CRYBB3/βB3-crystallin	gi|13928958	764	16	24,272.6	6.71
8	CRYBB3/βB3-crystallin	gi|9789594	325	15	24516.2	7.1
9	CRYAA/αA-crystallin	gi|202620	471	4	19273.5	5.91
10	CRYAA/αA-crystallin	gi|202622	736	2	19305.5	5.91
11	CRYAA/αA-crystallin	gi|202620	221	4	19273.5	5.91
12	CRYAA/αA-crystallin	gi|202622	438	2	19305.5	5.91
13	CRYAB/αB-crystallin	gi|57580	264	5	19945.3	6.84
14	CRYBA1/βA3-crystallin	gi|2338452	321	2	25317.9	5.8
15	CRYBA1/βA3-crystallin	gi|2338452	146	2	25.317.9	5.8
16	CRYAB/αB-crystallin	gi|30387800	231	5	20155.4	6.84
17	CRYGA/γA-crystallin	gi|124286843	336	5	21536	7.55
	CRYGB/γB/C-crystallin	gi|158138485	267	4	21530.9	7.55
	CRYGD/γD-crystallin	gi|14861862	106	9	21488.9	6.99
	CRYGE/γE/F-crystallin	gi|27545356	117	7	21591.9	7.11
§	CRYBA1/βA3-crystallin	gi|2338452	432	2	25317.9	5.8
§	CRYBB1/βB1-crystallin	gi|203623	262	9	28,013.5	8.59
	CRYBB1/βB1-crystallin	gi|203623	116	9	28,013.5	8.59
§	CRYBA2/βA2-crystallin	gi|27465611	431	6	22630.7	6.3
§	CRYGS/γS-crystallin	gi|157818045	225	6	14308.2	6.3

*Protein score: Protein Mscot score obtained by MALDI-TOF and tandem TOF/TOF mass spectrometry identification; a score >60 indicates that the protein identification can be considered successful. §Newly appeared protein spot.

### Western blot analysis

**Figure 5:**
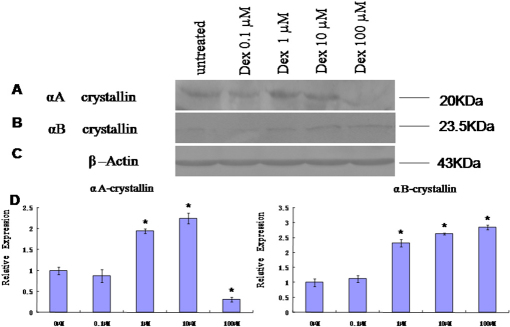
Protein levels of αA-crystalline and αB-crystallin. **A**: western blot of protein samples (60 mg) prepared from the normal and Dex-exposed lenses. The blots were probed with anti-αA-crystallin monoclonal antibodies. **B**: Blots probed with anti-αB-crystallin polyclonal antibodies FL-175. The approximate sizes of αA-crystallin and αB-crystallin are 20 kDa and 23.5 kDa, respectively. Imaging as described above, and densitometric scans for blot analysis were performed using NIH Image (ver.1.63) with values normalized to the β-actin signal for each sample and expressed as multiples of increase (or decrease) relative to control samples. The expression of αA- and αB- crystallin were significantly increased when lenses were exposed to 1 µM and 10 µM Dex (p<0.05, n=3). The expression of αA-crystallin was significantly decreased when lenses were exposed to 100 µM Dex (densitometric scans of blots were decreased; p<0.05, n=3). Data are the means of three western blot experiments, each performed in triplicate, and the pictures are representative.

To further verify the 2DE and modified spots results, two important crystallins: αA-crystallin and αB-crystallin, were analyzed by western blot. The three crystallins were selected based on their important roles in maintaining transparency of lenses. By western blot, as shown in Figure 5, αA-crystallin and αB-crystallin were significantly (p<0.05) increased when the lens was exposed to 1 µM Dex and 10 µM Dex. αA-crystallin significantly (p<0.05) decreased when the lens was exposed to 100 µM Dex.

### Inmmuno histology analysis on location of αA-crystallin and αB-crystallin

**Figure 6:**
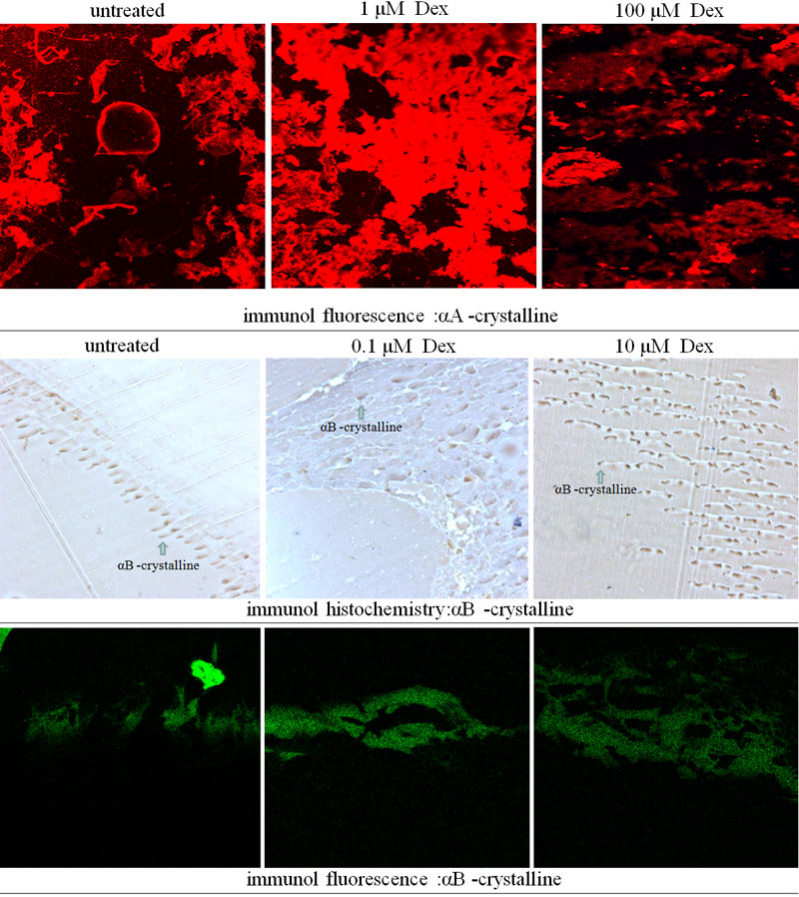
Histology of lenses without or with dexamethasone-exposed lenses. Whole lenses were cultured for 48 h without (control) or with dexamethasone. Lens sections were stained with primary antibodies(same with western-blot experiment) then stained with a second round of antibodies (HRP-conjuncted,Cy3 or FITC-conjuncted).

To characterize the effects of dexamethasone on cataract formation and location of αA-crystallin and αB-crystallin, organ-cultured rat lenses were examined histologically. The control lenses had normal cellular architecture and the lens fiber cells were regularly packed (Figure 6) 48 h after the culture with dexamethasone. After an incubation primer and second antibodies of αA-crystallin and αB-crystallin, figures of inmmuno histochemistry and inmmuno fluorescence showed that the quantities of αA-crystallin increased when the lens was exposed to 1 µM Dex, αB-crystallin increased when the lens was exposed to 10 µM Dex.

### The morphologic changes of lens fiber cells detected by electronic microscopy

**Figure 7:**
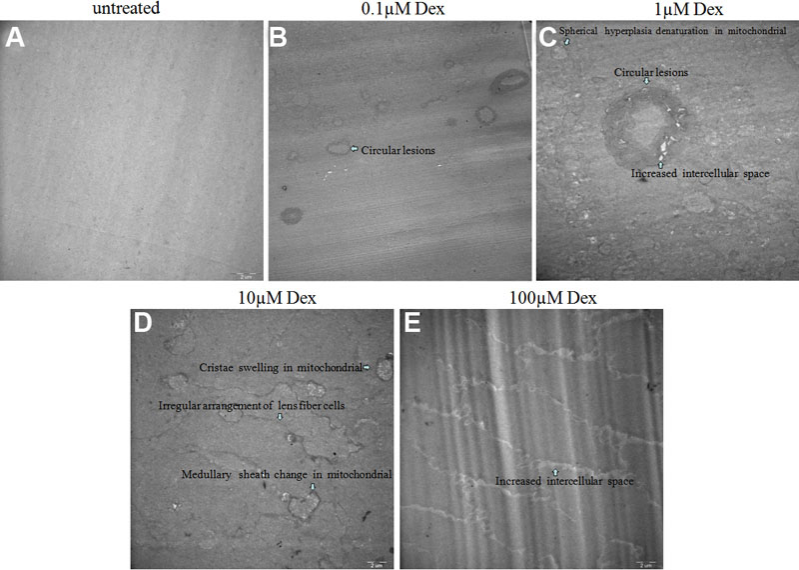
EM showed some changes in lens without or with Dex exposed lenses. **A**: Clear lens exposed to 0 µM Dex. **B**: Circular lesions were found located between the lens fiber cells. **C**: Showing increased intercellular space, circular lesions, spherical hyperplasia denaturation were found in mitochondrial of fiber cells. **D**: Showing irregular arrangement of lens fiber cells, cristae swelling and medullary sheath change in mitochondrial. **E**: Seriously increased intercellular space was found after the lens was exposed to 100 µM Dex.

As shown in Figure 7, we used EM to observe in more detail the morphologic changes at the cellular level. After the lens was exposed to 1–100 µM Dex, the fiber cells in the posterior lens displayed a disordered arrangement, and the tight contact between fiber cell membranes separated. Circular lesions were located between the lens fiber cells, or the fiber cells, but not intracellularly. We also found that in lenses exposed to 1–100 µM Dex, the degree of crack between lens fiber cells was consistent with the degree of lens cataract.

### Expressional changes of native crystallins

**Figure 8:**
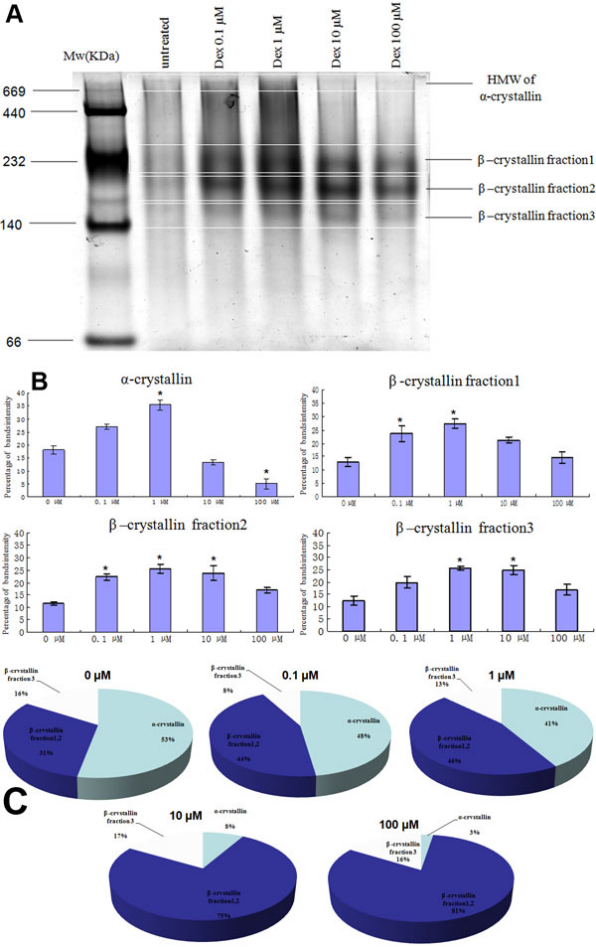
Native page of crystallins. **A**: Native page showed the bands of native α-crystallin isolated from lenses exposed to 0–100 µM Dex located on the top of the page and HMW of α-crystallin were larger than 700 kDa. Native β-crystallin located on the middle of the page. **B**: Expressional levels of α-crystallin increased after lens was exposed to 1 µM Dex, β-crystallin increased after lens was exposed to Dex (*<0.05 means significantly). **C**: Analysis of percentages of native crystallins showed percentage of α-crystallin gradually decreased and β-crystallin gradually increased with increasing concentrations of Dex.

We used native-page to analyze expressional changes of α-crystallin. Figure 8 showed the bands of native α-crystallin isolated from lenses located on the top of the page and indicated that HMW were larger than 700 kDa. Three β-crystallin fractions (1, 2, 3) were isolated from lenses located in the middle of the page and the molecular weights ranged from 140 kDa to 232 kDa.

## Discussion

α-, β-, and γ-crystallins are three major structural proteins contained in mammalian lenses. These structural proteins, by virtue of their specific structural interactions and high concentrations, are important to maintain the transparency of the lens. However, their relative importance in the development of lens opacity (induced by Dex) is not well understood. It also remains unclear how the relative mechanism of water insolubilization of lens crystallins during cataract development differs from the cataractous process induced by Dex. Some literature has suggested that a variety of posttranslational modifications cause aggregation and cross-linking of crystallins and lead to their water insolubilization. However, it is now believed that the development of lens opacity might involve mechanisms induced by more than one modification of crystallins.

In this study, seven major protein spots, including αA-crystallin, αB-crystallin, βA3-crystallin, βB2-crystallin, βA1-crystallin, βA4-crystalline, and βB1-crystalline, were identified in a 2DE map of normal rat lenses and cataractous lenses. The expression of αA-, αB-, βA3-, βB2-crystalline increased under various concentrations of Dex while βA1-, βA4- and βB1-crystallin were down regulated. Here, we discuss the crystallins that appear with abnormally high or low expression and their related changes.

Some groups have used two-dimensional electrophoresis (2DE) to study lenses from the aspect of proteomic analysis [16,17].We produced 2DE proteome maps of WI-US proteins in rat lenses after being exposed to 0–100 µM Dex; we then analyzed changes in the abundance of individual proteins and related changes in Dex-exposed lenses. These studies systematically investigated the varieties of modified proteins when a lens is exposed to varying concentrations of Dex, from 0 µM to 100 µM.

To ensure that the aggregates were non-disulfide-linked, the IPG strips were treated with 0.5% DTT and 4.5% iodoacetamide after the first dimension of isoelectric focusing (IEF) separation and before the second dimension. In the present study, we used 2DE to identify different levels of proteins in a direct comparison of normal and dexamethasone-exposed lenses. The 2DE technique provided a reliable display of proteomic differences between samples based on the multiplexing approach for accurate matching across gels. 2DE analysis identified 25 spots that represent four unique proteins’ expressions, increased on various concentrations of Dex and three proteins’ expression significantly decreased under concentrations of Dex. Because of four concentrations of Dex, we have provided for the first time the common changes and tendencies of some proteins in cataract lenses induced by Dex with different grades (Figure 1). The 2DE method identified which spots significantly increased or decreased in the Dex-exposed lenses.

The masses and measured isoelectric points (pIs) of rat crystallins suggest that the five new appearances of spots were: βA3-crystallin, two β-B1-crystalline protein spots, βA2-crystallin, and γS-crystallin.

### αA-crystallin

In the present study, the levels of αA-crystallin increased when lenses were exposed to 1 or 10 µM Dex compared with normal controls, and the western-blot figures showed decreased levels of αA-crystallin, which became more obvious as the lens was exposed to the highest concentration of Dex, perhaps because αA-crystallin gradually became water-insoluble-urea-insoluble or because the structure of it was destroyed and the antibody of αA-crystallin could not be combined with αA-crystallin effectively. A previous paper [17] reported that α-crystallin aggregates were approximately 600 kDa, and the γ-crystallin monomers of approximately 20 kDa molecular weight. Because we did not find obvious γ-crystallin (20 kDa), we could not be sure of native α-crystallin isolated from lenses exposed to 0–100 µM Dex located on the top of the page, and whether HMW of α-crystallin were larger than 700 kDa and native β-crystallin located on the middle of the page.

Although αA-crystallin increased under 1 or 10 µM Dex, figures of native-page showed that the percentage of α-crystallin gradually decreased when accompanied with increased concentrations of Dex; α-crystallin is an abundant protein in the eye lens of almost all vertebrates, reaching levels of up to 50% of water-soluble lens protein [18,19]. In our study, we also found that the percentage of α-crystallin was 53% in whole WI-US crystallins of normal lenses, but that it gradually decreased to 3% in whole WI-US crystallins after the lens was exposed to the highest concentration of Dex.

α-Crystallin belongs to the family of small heat-shock proteins (sHSP), and the α-crystallin domain is common to all the members of the sHSP superfamily [18,19]. Like other sHSP, α-crystallin functions like a molecular chaperone by suppressing the aggregation of other cellular proteins under various stress conditions [18,19].

In vitro, α-crystallin can protect β- and γ-crystallins and other proteins against denaturation and subsequent aggregation [20,21]. The mechanism of this protection involves binding of the partially denatured protein to a central region of an α-crystallin complex [22]. The α-crystallin may perform a similar function in vivo, binding to and protecting other crystallines against further denaturation [23]. In our study, we also found the expression of βA1-, βA4-, βB1-crystallin decreased under concentrations of Dex. After being attacked by Dex, the expressional levels decreased more than in normal controls, α-crystallin likely loses its chaperoning abilities, the remaining α-crystallin will probably be overwhelmed prematurely, thereby making damaged β-crystallin more prone to aggregated to WI-UI crystallins. The decreased expression of crystallins mentioned above are mainly because of the decreased expression of α-crystallin, which cannot protect the denaturation of them. The molecular chaperone properties of α-crystallin are believed to play a role in maintaining the transparency of the lens [23]. As expression levels of α-crystallin decreases, it may lose its chaperone abilities, thereby leading to a decrease in the ability to protect the denaturation of other proteins or crystallins when rat lenses are exposed to concentrations of Dex. Further, the electronic microscope showed that the intercellular space between lens fiber cells became larger, the degree of crack accompanied with increased concentration of Dex and mitochondrial were attacked to some extent when a lens was exposed to Dex.

We also found αA-crystallin became progressively and obviously acidified, phosphorylated in the lens WI-US fraction, with the phosphorylated form comprising one-third of the total αA when lenses were exposed to higher concentrations (1, 10, and 100µM) Dex. Phosphorylation of α -crystallins has already been well documented in rat lenses [24]. Further studies are required to determine which mechanism of α-crystallin phosphorylation predominates in rats. These studies are important, because phosphorylation of α-crystallin may cause dissociation of α-oligomers and reduction of chaperone-like activity [25]. This modification could cause the β- and γ-crystallin to partially unfold and further aggregate.

The expression of αA-crystallin further detected by western blot, immune fluorescence, and the results are consistent with the expressional levels detected by the 2-DE map.

### αB-crystallin

Based on previous reports, which suggested that αB-crystallin was important for a chaperone function, it also appears to exhibit anti-apoptotic functions. We studied the expressional levels of αB-crystallin after lenses were exposed to concentrations of Dex. Our data suggests the levels of αB-crystallin increased when lenses were exposed to higher concentrations of Dex compared with normal controls, perhaps this was a reaction to stress for lenses placed under Dex. Because the expressional levels of α-crystallin detected by native-page showed it gradually decreased, accompanied by the increasing concentrations of Dex, the lens could not escape the fate of other crystallins being attacked and denatured, and the overall ability to protect other proteins decreased.

The expression of αB-crystallin was further detected by western blot, immuno histochemistry, and immune fluorescence, and the results are consistent with the expressional levels detected by 2DE mapping.

### β-crystallines

The present literature suggests that β-crystallin oligomers exist and play a critical role in maintenance of lens transparency [26].

Excessive accumulation of modified β-crystalline in vivo may disrupt normal protein–protein interactions, diminishing their stability and thus contributing to the accumulation of insoluble β-crystalline during cataract development [27]. In our experiment, we also found that new protein spots appeared, compared with a normal lens, with increased concentrations of Dex: ßA3-, ßB1- (alkalization form), ßA2-, ßA1-, ßA4-, and ßB1-crystallins also experienced a decrease with increasing concentrations of Dex. This significant loss of ßA1-, ßA4-, and ßB1- crystallins may be more related to its greater tendency to undergo WI-UI during lens opacity. The figure of the native-page showed the percentages of β-crystallin gradually increased with increased concentrations of Dex, maybe because of newly appeared β-crystallin protein spots.

To detect the changes of lens fiber cells and the junctional condition of them, we further used EM to observe in more detail the morphologic changes at the cellular level. After a lens was exposed to 1–100 µM Dex, the fiber cells in the posterior lens displayed a disordered arrangement, and the tight contact between fiber cell membranes separated. Circular lesions were located between the lens fiber cells, or the fiber cell and capsules, but not intracellularly. We also found that when a lens was exposed to 1–100 µM Dex, the degree of crack between lens fiber cells was more serious depending on the degree of lens cataract. We speculated that this phenomenon may be caused by decreased normal β-crystallins and the appearance of new β-crystallin protein spots, which disorder the normal arrangement of lens fiber cells so that inter-cellular space became enlarged and the crack became more serious with increasing concentrations of Dex.

### γ**-**crystallins

Newly appeared protein spots: γS-crystallin appeared after the lens was exposed to 10 µM and 100 µM Dex. γS-crystallin, the concentration of which rapidly increases with age of rat [28]. In our study, we also found newly γS-crystallin protein spots appeared when the lens was exposed to a high concentration of Dex. Because γ-crystallin could not be focused and divided well in a WI-US fraction, we could not compare the expression levels of γA- and γE/F-, respectively.

Some previous reports [29,30] have indicated that the incidence of cataract formation is higher in the lenses of patients given prednisolone in eye drops than in those who receive it systemically, suggests that glucocorticoid directly affects the pathogenesis of a lens. The changes in the relative abundance of crystallins would be expected to significantly alter the properties of lens fibers of the dexamethasone exposed lens. The gradually diminishing native α-crystallin, decreased expression of β-, βA1-, βA4-, and βB1- may favor dehydration and further insolubilization with a higher index of refraction. In the future, we should study the structural changes of crystallins when lenses are exposed to varying concentrations of Dex. During glucocorticoid-induced cataract development, our results showing multiple crystallins may be more vulnerable to glucocorticoid stress because of diminished important roles. The possible mechanisms are needed to further investigate.

#### Conclusions

In conclusion, this study of rat lenses after being exposed to increased concentrations of Dex provides baseline data that will facilitate the variable expressional levels and analysis of modified WI-US crystallins appearing in glucocorticoid induced cataractous rat lens. We performed 2DE gel separation coupled with TOF-MS/MS analysis of in-gel digests. The 2DE maps produced by identification of proteins by TOF-MS/MS analysis can be directly compared with similar 2DE gels run in other laboratories. Related and comprehensive analysis of these modifications in cataractous rat lenses may provide the necessary information to model how these alterations contribute to insolubilization and light scatter. The information is particularly useful in future studies to more thoroughly examine glucocorticoid-induced cataract-specific modifications in rat crystallins, when compared with similar analysis of human crystallins based on several studies [31]. Our analysis is important, because similar modifications may be a cause of human cataracts.
